# Effectiveness of sanitization protocols in removing or reducing parasites from vegetables: A systematic review with meta-analysis

**DOI:** 10.1371/journal.pone.0290447

**Published:** 2023-09-01

**Authors:** Cláudio Márcio de Medeiros Maia, Karla Suzanne Florentino da Silva Chaves Damasceno, Larissa Mont’Alverne Jucá Seabra, Gabriela Chaves, Lívia Maria da Costa Dantas, Francisco Canindé de Sousa Júnior, Cristiane Fernandes de Assis

**Affiliations:** 1 Nutrition Postgraduate Program, Center for Health Sciences, Federal University of Rio Grande do Norte, Natal, Brazil; 2 Myant INC, Research and Development, Toronto, Canada; 3 Department of Pharmacy, Center for Health Sciences, Federal University of Rio Grande do Norte, Natal, Brazil; Eduardo Mondlane University: Universidade Eduardo Mondlane, MOZAMBIQUE

## Abstract

**Background:**

Parasitic contamination in vegetables is a reality in several countries and a challenge for food safety. The risk of consumption usually raw, associated with failures in good practices of production, transportation, and preparation further increase the possibility of ingesting contaminated food. Given this, a systematic review was carried out to scientifically demonstrate the effectiveness of sanitization protocols in the parasitic decontamination of plants.

**Methods:**

This review was conducted following the guidelines of the Cochrane Manual, being registered in the PROSPERO protocol base (CRD42020206929) and reported according to the PRISMA 2020 statement. The review evaluated studies published in the MEDLINE, Embase, Web of Science, FSTA, LILACS, and AGRIS databases, as well as manual searches of related articles, references, and theses and dissertations directories. The meta-analysis was performed using the Revman 5 software program, the bias assessment used the Robins I Tools with some adaptations, and the quality of the evidence was evaluated using GRADE.

**Discussion:**

The review included a total of 31 studies, most of which were carried out in countries with a high incidence of plant parasites, such as Brazil and Iran. Interventions combined with 200ppm chlorination preceded by brushing, rinsing, or immersion in detergent showed the greatest efficiency in parasitic decontamination. Despite the high heterogeneity and risk of bias in the primary studies, this review can inspire the planning of new studies which observe the critical and methodological evaluation for research in the field of food safety.

## 1. Introduction

Parasitic diseases are among the most frequent in the world and are a challenge for sanitary control in several developing countries [1]. The contamination of plants with parasites was recently recognized as a global threat and a risk alert for transmitting protozoa and helminths in the propagation of outbreaks [2, 3].

In this context of increased globalization in the food supply, the danger of introducing pathogenic agents into new geographic areas occurs by importing products and inputs from endemic areas [4]. This risk is further aggravated by the mostly raw consumption of these foods, which increases the epidemiological role of vegetables in the transmission of food-borne parasitic zoonoses [5].

Diseases caused by parasites transmitted in food (food-borne parasites—FBPs) are directly linked to several factors such as poor management conditions in agriculture, the use of organic fertilizer rich in parasites, contaminated irrigation water, unregulated use of environmental resources, as well as eating habits and cultural aspects of the population [6]. According to Mennerat et al. [7], the increase in the practice of intensive agriculture based on aggressive exploitation of the land strengthens the development of rapid life cycles of the parasite, facilitating the spread of diseases.

In systematic reviews that evaluated the global prevalence of parasites in fruits and vegetables, it was shown that the estimated occurrence of helminths in vegetables is 31% (95%CI (Confidence Interval) = 26%–37%) and 20% for protozoa (95%CI = 16%–24%). However, these values are much higher in underdeveloped or developing regions with percentages easily exceeding 50% [8, 9].

Considering the relevance of this topic, this study aims to carry out a systematic review of hygiene protocols for removing parasites in vegetables to evaluate the scientific evidence that improves hygiene practices and guarantees better quality of these foods.

## 2. Materials and methods

This review had its protocol registered in the PROSPERO database (International Prospective Register of Systematic Reviews), with registration number CRD42020206929 and is reported following the guidelines provided in the PRISMA 2020 guide (Preferred Reporting Items for Systematic Review and Meta-Analyses) described in **S1 File** [10]. The protocol of this study was also published [11], detailing the methodological process of this review and promoting greater transparency in the research.

### 2.1 Inclusion and exclusion criteria

The eligibility criteria for the study were defined using the PICOT classification (Population, Intervention, Comparator, Outcomes, and Study Types) as a tool to guide the research and formulate the search strategy. We defined the population (P) as studies that reported parasitic analyzes in plants before and after some intervention process. This intervention process (I) could be any sanitization agent (chemical, physical, or associated). In addition, the studies should present a comparator (C) of unhygienic samples or artificial contamination, the removal or reduction of parasitic forms adhered to the plants as an outcome (O), and comparative studies between samples with one or more intervention processes as the study type (T). Based on these definitions, structured terms were created to compose the search strategy. The primary search strategy and the modifications to meet the specificities of each database search syntax are detailed in **S2 File**.

### 2.2 Article selection and data extraction

The screening stage was carried out by two evaluators independently who indicated whether or not the study met the acceptance criteria by reading the titles and abstracts. A third reviewer decided on its inclusion in cases of disagreement.

Data were extracted in the same way with the completion of an Excel spreadsheet with data on methodology, number of samples, cleaning procedure, time, statistical data, and outcomes found. The two stages were carried out without any interference or contact between the reviewers to maintain transparency and avoid influence in the decision-making process.

### 2.3 Quality evaluation

The qualitative analysis of the articles was performed by assessing the risk of bias according to the Cochrane Robins I evaluation methodology [12] with some modifications to adapt to food analysis studies. This assessment was categorized into 5 domains: selection bias, sample bias, performance bias, detection bias, and reporting bias, according to **S3 File**.

A pilot analysis was initially conducted among raters to ensure that raters could apply the criteria consistently. Again, this step was independently performed by two evaluators, and disagreements between researchers were decided by a third evaluator.

### 2.4 Data synthesis and statistical analysis

The studies were evaluated by the parasite reduction percentage for the main outcome, and by the odds ratio value calculated by meta-analysis performed by the Review Manager software program (RevMan version 5.4).

We evaluated the heterogeneity using i-square statistics (I^2^) and the chi-squared test (Chi^2^). For significance values of p ≤ 0.10 and magnitude (I^2^ ˃ 50%), we concluded to conduct a qualitative synthesis of the results from the percentage values (**S4 File**), because the high heterogeneity decreases the robustness of the isolated findings of the meta-analysis [13, 14].

We performed a sensitivity assessment of subgroups to assess heterogeneity (**S6 File**). Finally, the quality of evidence from the systematic review was assessed using GRADE, which establishes the recommendation strength of the findings for decision-making [13].

## 3. Results

The initial search took place on October 16, 2020, and 1,380 references were found; however, after removing duplicate articles and screening the titles and abstracts, 49 publications were evaluated for meeting the inclusion criteria, with 24 studies selected by the initial search.

**Fig 1:**
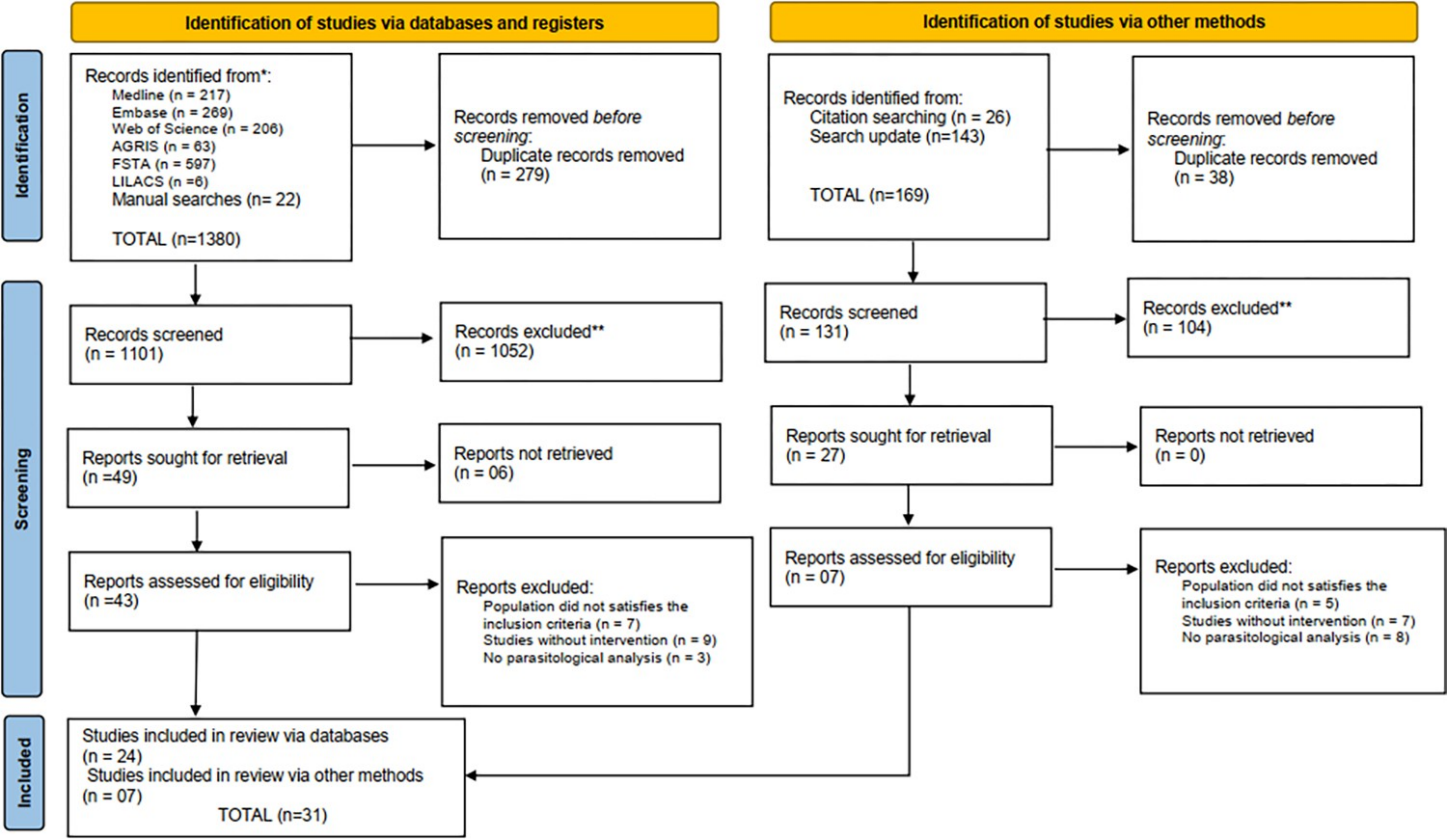
Prisma 2020 flowchart.

As the initial search was performed two years earlier, an update of the search for the databases and a manual search for citations of included articles was carried out. This update took place on May 20, 2022, following the same steps as the previous search, with 07 more publications being added to the 2020 searches, resulting in a total of 31 articles included. This entire study selection process is presented in **Fig 1** according to the PRISMA flowchart 2020 [10].

### 3.1 Characterization of the included studies

Of the 31 studies included, most were conducted in underdeveloped countries in Latin America, Africa, and the Middle East (83.9%), with Iran (22.6%) and Brazil (35.5%) having the highest number of publications.

**Fig 2:**
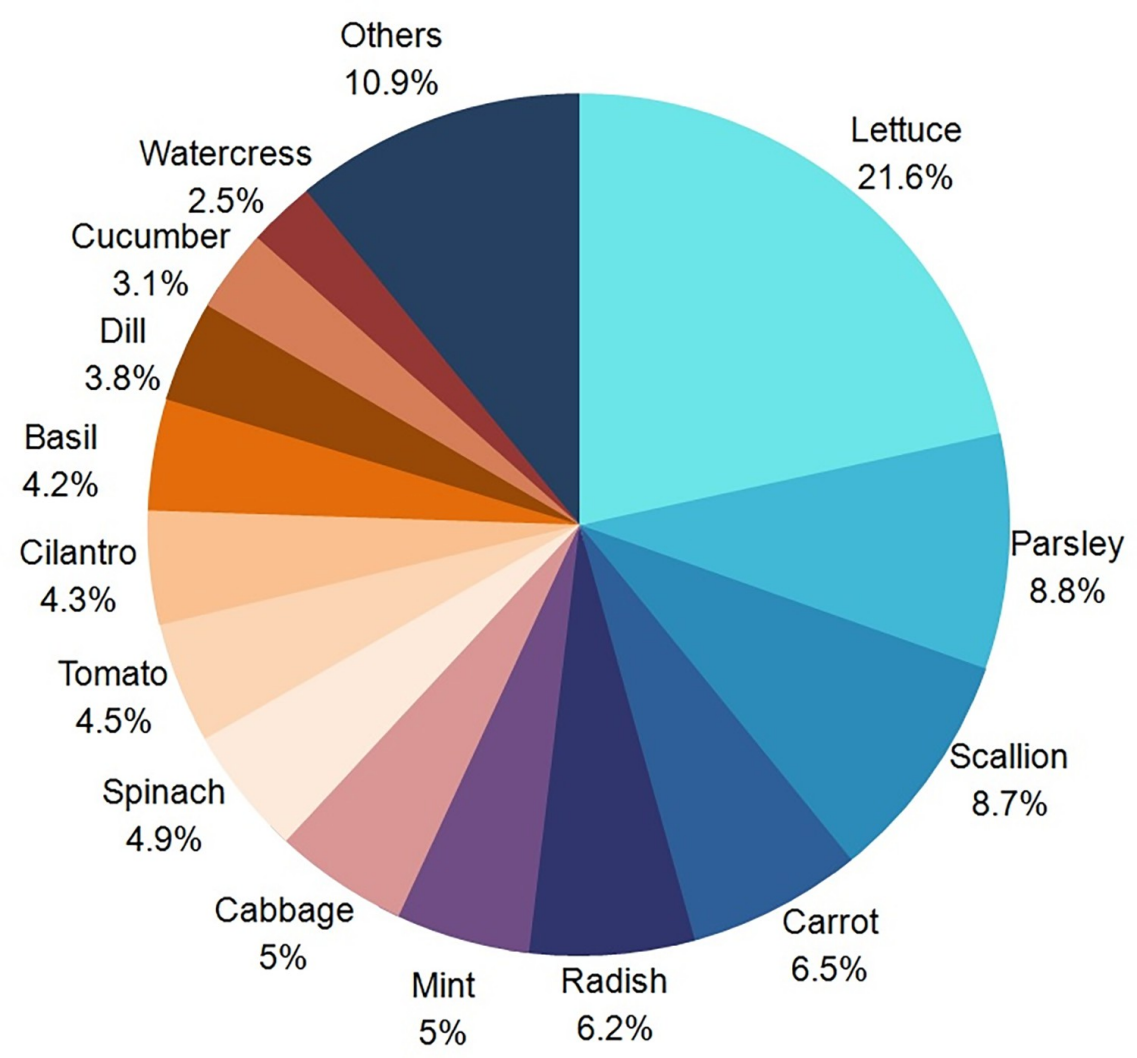
Percentage of the main foods searched.

Among the studies participating in this review, a total of 4,607 samples of 24 different types of vegetables were evaluated. Lettuce was the most analyzed product with 21.1% of the total vegetables surveyed, followed by parsley (8.5%) and chives/green onions (8.0%), as shown in **Fig 2**.

The 31 selected references presented a total of 112 interventions because several studies presented comparisons between more than one cleaning process. Of these hygiene methods, 62 of them were exclusively performed using chemical products, while 22 used exclusively physical treatments, and the remaining 28 combined physical and chemical processes.

**Fig 3:**
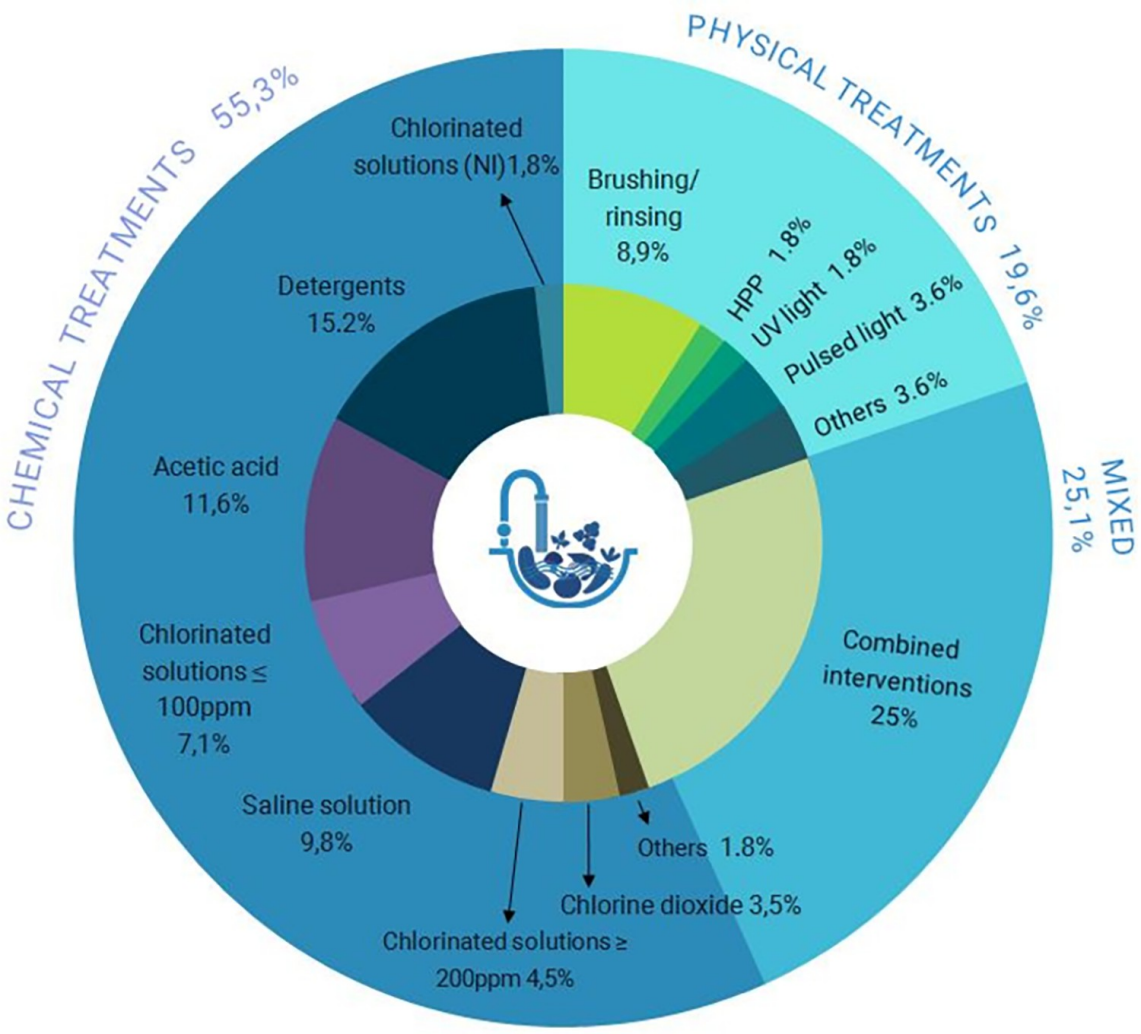
Percentage of intervention processes applied.

Chlorine was the most common product among the chemical reagents used, being used in 30% of the total interventions applied, followed by acetic acid and detergent, both with 18%. Mixed treatments were procedures that mixed steps of chemical and physical treatments, and there were no interventions with more than one physical procedure combined (**Fig 3**).

### 3.2 Publication bias

**Fig 4:**
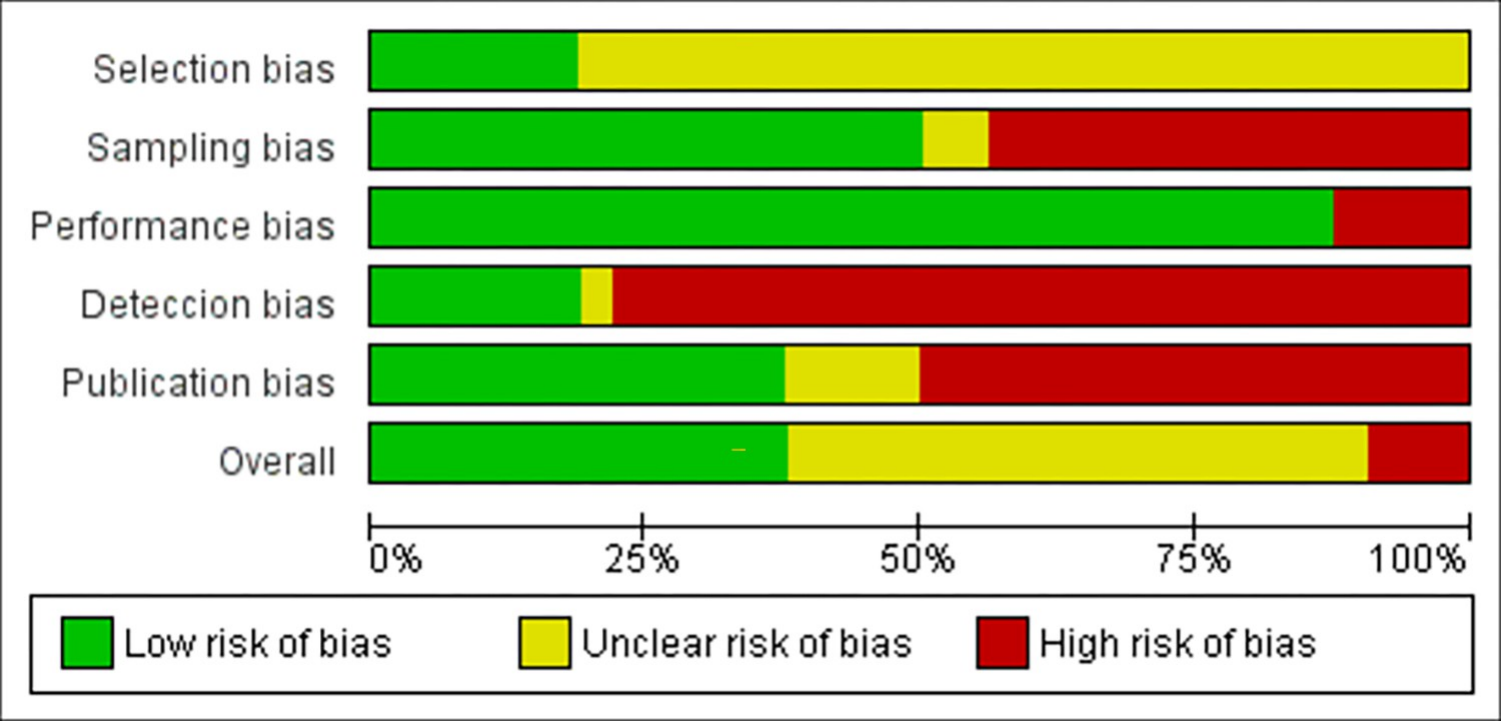
Overall graph of the bias assessment of the included studies.

The studies mostly presented a level of uncertainty considered moderate. Among the main points evaluated by the reviewers, the domains of detection bias, publication bias, and sampling presented the highest levels of uncertainty, according to **Fig 4**.

### 3.3 Meta-analysis of the subgroups

The primary studies reported rates of reduction or absence of parasitic forms in vegetables in much diversified measurement units, such as the number of contaminated samples, parasite count (absolute number and logarithm), excystation rate, infectivity rate, or parasite inactivation percentage, among others.

**Fig 5:**
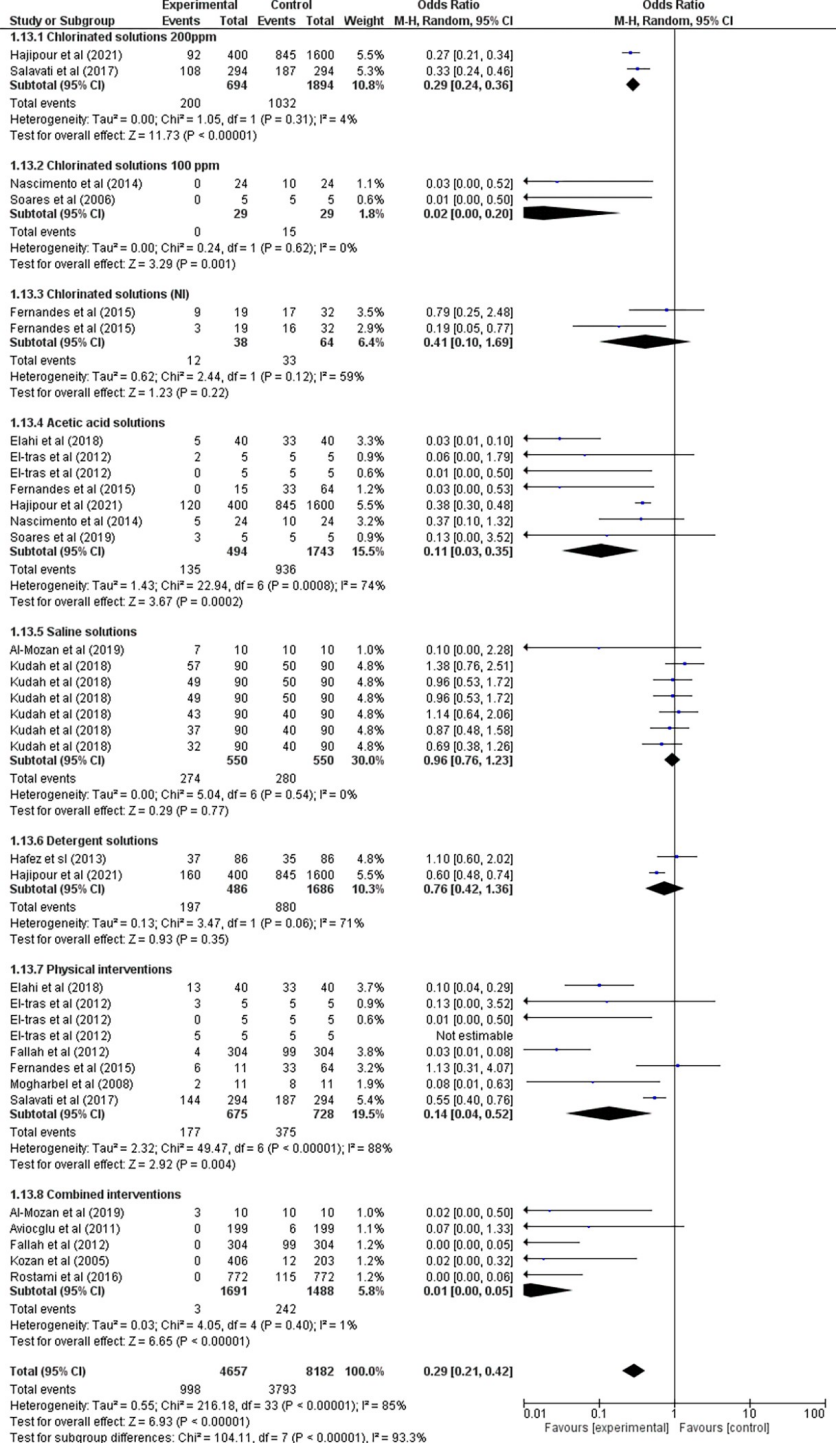
Meta-analyses of studies by the Mantel-Haenszel (MH) method.

**Fig 6:**
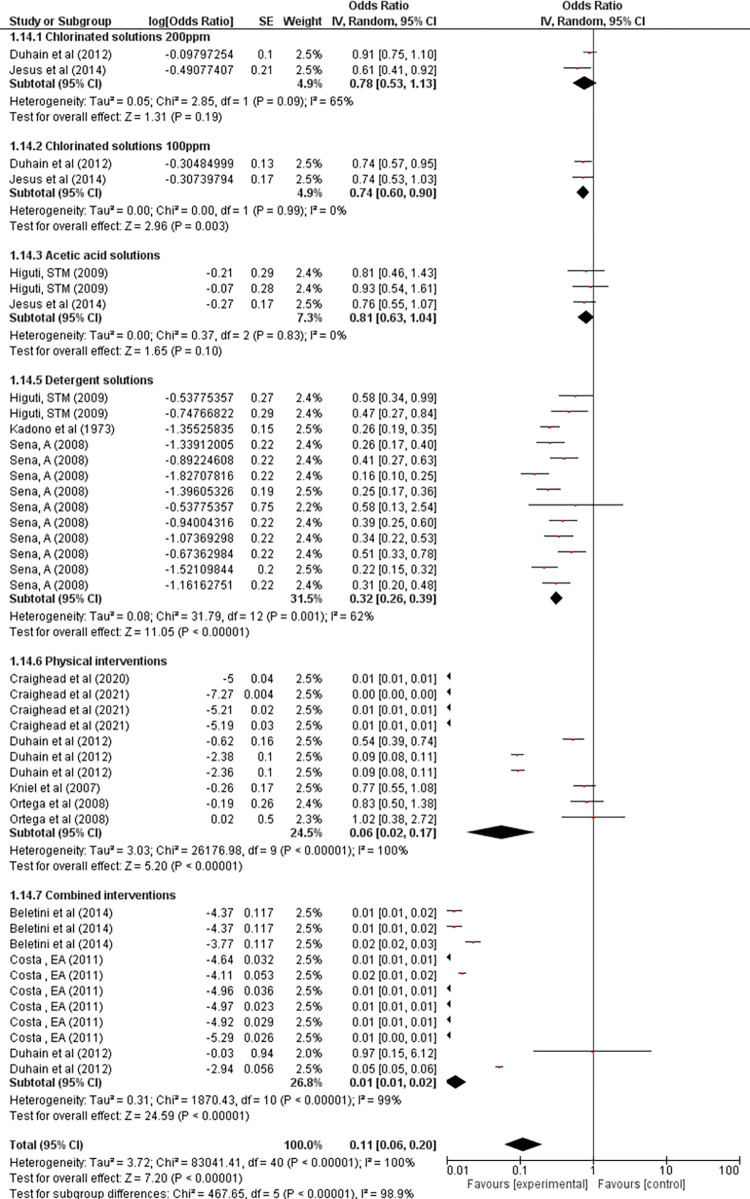
Meta-analyses of inverse variance studies (IV).

All this diversity of reported outcomes resulted in in a meta-analysis with high heterogeneity (Heterogeneity: (P < 0.00001); I^2^ = 93%), making an overall assessment impossible. Thus, the results are presented in subgroup and sensitivity analyses (**Figs 5 and 6 and S6 File**), in addition to the descriptive data of the sanitizing effect adjusted by the parasite reduction percentage, as established in the protocol of this review and detailed in **S4 File.**

The meta-analyses were divided into two different statistical methods as the measurement units were not compatible with a single grouping. Thus, outcomes with dichotomous results were evaluated using the Mantel-Haenszel (MH) method, which determines the odds ratio between post-intervention contaminated samples and control samples. The continuous outcomes were calculated from the Inverse of Variance (IV) that uses the standard error and the logarithm of the odds ratio (OR) for valuation in the graph.

## 4. Discussion

The close relationship between the number of prevalence studies and control and sanitation studies reflects the importance that parasitic contamination has for some endemic regions of the planet. Thebo et al. [15] evaluated that greater use of untreated urban wastewater on agricultural land is observed in Caribbean, Latin American, Middle Eastern (mainly Iran), and Asian countries, with the cultivation of vegetables being the most frequent type of crop in periurban regions. This combination of parasite prevalence in plants and poor sanitary conditions in cultivation suggests the strong influence that inadequate agricultural practices and lack of structure have on the incidence of cases.

Among the types of samples surveyed, it is interesting to observe the predilection for leafy vegetables in the analyses, such as lettuce, parsley, and spinach (**Fig 2**). This fact may be explained by prevalence studies, which revealed that lettuce is the vegetable with the highest contamination [16–20], and this probably influences the choice of this type of vegetable in the analyzes.

The highest risk rates in the bias assessment were observed in the detection domain, which is due to the strict use of microscopy without concern for reducing the analyst’s bias. Given that the microscopic method already intrinsically has a high level of subjectivity and has methodological fragility when performed without randomization, blinding, random generation of numbers, or by more than one analyst, as the analysis with prior knowledge of the treatment performed on the sample generates risk for bias in the result.

Another item that drew attention was related to publication bias. Many studies carried out the parasite detection step using the liquid from the extraction phase as a sample, however, this procedure also contains a high risk of bias, as this type of sample does not reflect a fully sanitized vegetable, which is the sample after passing through the sanitization process and then being submitted to the analytical process.

The artificial contaminations which used a very low number of samples influenced the high result of the sample bias value, as they have a greater uncertainty of results. Individual bias assessments are described in **S5 File**.

In analyzing the meta-analysis graphs, we see in **Fig 5** that the combined interventions had the greatest sanitizing effect for removing parasites in vegetables (OR = 0.01 95%CI: 0.00–0.05), meaning that there is a ratio of 99.99% less chance of finding parasites in this type of intervention when compared to non-clean vegetables.

The 200ppm chlorine solutions showed a smaller effect (OR = 0.29 95%CI: 0.24–0.36) and the chlorine solutions ≤ 100ppm contained studies with a very low sample number; therefore, despite the mean OR value being lower than the 200ppm chlorine solutions, the confidence interval is wider, which results in greater uncertainty of results (OR = 0.02 95%CI: 0.00–0.05).

Interventions using saline solutions have a heterogeneity of 0%; however, all outcomes exceed the central null line (OR = 0.02 95%CI: 0.76–1.23), meaning that it is not possible to state that this type of intervention promotes efficient action in removing parasites compared to non-sanitized samples. The other interventions calculated by Mantel-Haenszel obtained heterogeneity rates above 50% and therefore presented invalid OR results.

These meta-analytical data corroborate the sanitation percentage charts (**S4 File)** by demonstrating that cleaning with chlorine alone is not as efficient in parasite removal, which is a fact that is reported by some studies regarding the difficulty in acting on parasite adhesion and the interference of the oxidizing action in the presence of organic matter [4, 21, 22]. This suggests that rinsing, brushing, or concomitant use of another product, such as a detergent solution, favors contact of the parasite with chlorine and facilitates its removal from the food.

Regarding **Fig 6**, we only had valid results for interventions with acetic acid solutions (OR = 0.81 95%CI: 0.63–1.04) and chlorine solutions ≤ 100ppm (OR = 0.74 95%CI: 0.60–0.90), but both present OR results which are much lower than the other interventions and come close to the central null line.

It is important to highlight that the physical interventions calculated by IV that reflect treatments by U.V light, pulsed light, and atmospheric plasma present excellent decontamination results, as well as the combined interventions of **Fig 6**; however, as they are very heterogeneous groups, they did not obtain valid OR results, as the heterogeneity was greater than 50%, requiring that more comparative studies be carried out to prove the sanitizing effect of these interventions in different conditions.

Even so, some studies could not be included in the meta-analysis, as they did not contain enough information, and even after contacting us by email, we did not get a response regarding the requested information, so these studies will be represented in **S4 File** for descriptive analysis.

**S7 File** presents the GRADE assessment for this systematic review, and the outcomes are classified in a table by sanitization type. Only sanitization with chlorine solutions above 200ppm obtained a moderate confidence level, which means that it is likely that the true effect is close to the estimate found; however, there is the possibility that future work will substantially modify the magnitude of the estimate of this effect, and may even modify the estimate [13].

The inherent limitations of conducting studies with a high level of bias, in addition to the inconsistencies caused by the high heterogeneity between studies and the imprecision of studies with very wide confidence intervals were the factors that most contributed to lowering confidence in the GRADE assessment.

To increase the sensitivity of this systematic review, the search strategy and the databases used were very comprehensive, in addition to non-restrictive acceptance criteria regarding time, language, and use of different methodologies, which focused on grouping studies with great methodological variability, and therefore the recommendation strength and reliability of the outcomes found had low levels.

## 5. Conclusions

Most of the selected studies were carried out in underdeveloped or developing countries which have high parasite prevalence rates in plants. Lettuce was the most researched food, with treatments using chemical reagents being the most common.

The evidence synthesis showed that decontamination by combined interventions using chlorine solutions followed by brushing, rinsing, or previous immersion in detergents presented the best results for parasite removal in vegetables. However, the selected studies showed high heterogeneity with a moderate risk of bias, which lowered the level of quality of the findings.

It is important to note that although the quality of evidence in this review was evaluated as moderate to low, the outcomes found in this study may contribute to decision-making regarding the best hygiene protocol for vegetables and inspire planning for future studies, observing the critical analysis and methodological approaches addressed in this review.

## Supporting information

S1 File Prisma 2020 checklist. (DOCX)

S2 File Table of search strategies. (DOCX)

S3 File Bias evaluation table. (XLSX)

S4 File Table of the parasitic reduction percentage. (DOCX)

S5 File Results of the bias assessment per study. (PNG)

S6 File Sensitivity analysis of subgroups. (DOCX)

S7 File Evidence certainty assessment (GRADE). (DOCX)
