# Supplementary material for: Effectiveness of sanitization protocols in removing or reducing parasites from vegetables: A systematic review with meta-analysis
Source: PLoS One. 2023 Sep 1;18(9):e0290447. doi: 10.1371/journal.pone.0290447 (PMC10473522; doi:10.1371/journal.pone.0290447)
Supplement: S2 File — (DOCX) [file pone.0290447.s002.docx]

| Supplementary Material 2 - Search strategies | | | |
| --- | --- | --- | --- |
| First search: 10.16.2020 Update search:05.20.2022 | | | |
| Databases | **Search strategy** | **Number of records** | **Fields** |
| Ovid MEDLINE | \| 1 \| exp Lettuce/ \| \| --- \| --- \| \| 2 \| vegetables.mp. \| \| 3 \| fresh produce.mp. \| \| 4 \| Plants, Edible.mp. \| \| 5 \| leafy vegetables.mp. \| \| 6 \| 1 or 2 or 3 or 4 or 5 \| \| 7 \| Parasites/ or parasit*.mp. \| \| 8 \| exp Oocysts/ \| \| 9 \| exp Helminths/ \| \| 10 \| Parasite Egg Count/ \| \| 11 \| parasite examination.mp. \| \| 12 \| intestine parasite.mp. \| \| 13 \| 7 or 8 or 9 or 10 or 11 or 12 \| \| 14 \| exp Disinfection/ \| \| 15 \| hypochlorite.mp. or Hypochlorous Acid/ \| \| 16 \| Peracetic Acid/ \| \| 17 \| Hydrogen Peroxide/ \| \| 18 \| Detergents/ \| \| 19 \| Chlorine/ \| \| 20 \| disinfection agent.mp. \| \| 21 \| Decontamination/ \| \| 22 \| chemical agent.mp. \| \| 23 \| Sanitation/ \| \| 24 \| Anti-Infective Agents/ \| \| 25 \| 14 or 15 or 16 or 17 or 18 or 19 or 20 or 21 or 22 or 23 or 24 \| \| 26 \| 6 and 13 and 25 \| | 217 | title and  abstract |
| Web of Science | \| 1 \| TS=(VEGETABLES) \| \| --- \| --- \| \| 2 \| TS=(LETTUCE \| \| 3 \| TS=("FRESH PRODUCE") \| \| 4 \| TS=("EDIBLE PLANTS") \| \| 5 \| TS=("LEAFY VEGETABLES") \| \| 6 \| TS=("SALAD*") \| \| 7 \| #1 OR #2 OR #3 OR #4 OR #5 OR #6 \| \| 8 \| TS=("PARASIT*") \| \| 9 \| TS=OOCYST \| \| 10 \| TS = HELMINTH* \| \| 11 \| TS = "EGG COUNT \| \| 12 \| TS = "PARASITE EGG COUNT" \| \| 13 \| TS = "PARASITE EXAMINATION" \| \| 14 \| TS = "PARASITE INTESTINE" \| \| 15 \| TS = "ENTAMOEBA" \| \| 16 \| #8 OR #9 OR #10 OR #11 OR #12 OR #13 OR #14 OR #15 \| \| 17 \| TS = "Disinfection" \| \| 18 \| TS = "hypochlorite" \| \| 19 \| TS = "Hypochlorous Acid" \| \| 20 \| TS = "Peracetic Acid" \| \| 21 \| TS = "Hydrogen Peroxide" \| \| 22 \| TS = "Detergent" \| \| 23 \| TS = "Chlorine" \| \| 24 \| TS = "disinfection agent" \| \| 25 \| TS = "Decontamination" \| \| 26 \| TS = "chemical agent" \| \| 27 \| TS = "Sanitation" \| \| 28 \| TS = "Wash" \| \| 29 \| TS = "Clean*" \| \| 30 \| TS = "Anti-Infective Agent*" \| \| 31 \| TS = "Ozone" \| \| 32 \| TS = "Ultraviolet" \| \| 33 \| TS = "Food safety" \| \| 34 \| TS = "Food quality" \| \| 35 \| TS = "Food analysis" \| \| 36 \| TS = "Food contamination" \| \| 37 \| TS = "Food parasitology" \| \| 38 \| TS = "Disinfectants" \| \| 39 \| #17 OR #18 OR #19 OR #20 OR #21 OR #22 OR #23 OR #24 OR #25 OR #26 OR #27 OR #28 OR #29 OR #30 OR #31 OR #32 OR #33 OR #34 OR #35 OR #36 O #37 OR #38 \| \| 40 \| #7 AND #16 AND #39 \| | 206 | title,  abstract  and  keywords |
| Embase | 'leafy vegetable'/exp OR 'romaine lettuce'/exp OR 'iceberg lettuce'/exp OR 'basil'/exp) AND ('disinfection'/exp OR 'sanitation'/exp OR 'disinfectant agent'/exp OR 'decontamination'/exp OR 'chemical agent'/exp) AND ('food quality' OR 'quality control procedures' OR 'food control' OR 'food analysis' OR 'parasitology' OR 'parasites' OR 'parasites egg count' OR 'parasite examination') | 269 | Title or  abstract |
| FSTA | ("Lettuce" OR "Vegetables" OR "fresh produce" OR " Edible Plants" OR "leafy vegetables" OR "Salads") AND (“Parasites” OR “Parasit*” OR “Oocysts” OR “Helminths” OR “Egg Count” OR “Parasite Egg Count” OR “parasite examination” OR “intestine parasite” OR “Entamoeba”) AND (“Disinfection” OR “hypochlorite” OR “Hypochlorous Acid” OR “Peracetic Acid” OR “Hydrogen Peroxide” OR “Detergents” OR “Chlorine” OR “disinfection agent” OR “Decontamination” OR “chemical agent” OR “Sanitation” OR “Wash” OR “Clean” OR “Anti-Infective Agents” OR “Ozone” OR “Ultraviolet” OR “Food safety” OR “Food quality” OR “Food analysis” OR “Food contamination” OR “Food parasitology” OR “Disinfection”) | 597 | Title or  abstract |
| Lilacs | ("Lettuce" OR "Vegetables" OR "fresh produce" OR " Edible Plants" OR "leafy vegetables" OR "Salads") AND (“Parasites” OR “Parasit*” OR “Oocysts” OR “Helminths” OR “Egg Count” OR “Parasite Egg Count” OR “parasite examination” OR “intestine parasite” OR “Entamoeba”) AND (“Disinfection” OR “hypochlorite” OR “Hypochlorous Acid” OR “Peracetic Acid” OR “Hydrogen Peroxide” OR “Detergents” OR “Chlorine” OR “disinfection agent” OR “Decontamination” OR “chemical agent” OR “Sanitation” OR “Wash” OR “Clean” OR “Anti-Infective Agents” OR “Ozone” OR “Ultraviolet” OR “Food safety” OR “Food quality” OR “Food analysis” OR “Food contamination” OR “Food parasitology” OR “Disinfection”) | 6 | Words |
| Scopus | ('lettuce/exp' OR 'leafy vegetables/exp') AND ('parasit*' OR 'oocyst*' OR 'egg*' OR 'helminth' OR 'egg count') AND ('disinfection' OR 'ozone' OR 'hypochlorite' OR 'chlori*' OR 'peracetic acid' OR 'detergent' OR 'decontamination' OR 'wash*' OR 'sonic' OR 'sanit*' OR 'clean*') AND ('food quality/exp' OR 'food safety/exp' OR 'food hygiene/exp' OR 'food contamination/exp' OR 'food analysis' OR 'efficiency' OR 'efficacy' OR 'effects') | 3 | All fields |
| AGRIS | ('lettuce/exp' OR 'leafy vegetables/exp') AND ('parasit*' OR 'oocyst*' OR 'egg*' OR 'helminth' OR 'egg count') AND ('disinfection' OR 'ozone' OR 'hypochlorite' OR 'chlori*' OR 'peracetic acid' OR 'detergent' OR 'decontamination' OR 'wash*' OR 'sonic' OR 'sanit*' OR 'clean*') AND ('food quality/exp' OR 'food safety/exp' OR 'food hygiene/exp' OR 'food contamination/exp' OR 'food analysis' OR 'efficiency' OR 'efficacy' OR 'effects') | 63 | Publications and datasets |
