# Supplementary material for: Effectiveness of sanitization protocols in removing or reducing parasites from vegetables: A systematic review with meta-analysis
Source: PLoS One. 2023 Sep 1;18(9):e0290447. doi: 10.1371/journal.pone.0290447 (PMC10473522; doi:10.1371/journal.pone.0290447)
Supplement: S4 File — (DOCX) [file pone.0290447.s004.docx]

# Supplementary material 4 - Table of the parasitic reduction percentage per study and type of treatment.

| TYPE OF TREATMENT | STUDY | INTERVENTION | REDUCTION % |
| --- | --- | --- | --- |
| Chlorine solutions≥ 200ppm | Duhain et al. [1] | Immersion in 200ppm chlorine solution | 1% |
|  | Jesus et al. [2] | Immersion in 200ppm sodium hypochlorite solution | 16.5% |
|  | Silva [3] | Immersion in 200 to 250ppm bleach solution | 0% |
|  | Salavati et al. [4] | Immersion in 200ppm calcium hypochlorite solution | 42.1% |
|  | Hajipour et al. [5] | Immersion in 200ppm calcium hypochlorite solution | 98.6% |
| Chlorine solutions≤ 100ppm | Silva [3] | Soaking in 70ppm Pury Vita solution | 0% |
|  | Silva [3] | Immersion in 80ppm Useclor solution | 0% |
|  | Silva [3] | Immersion in 25ppm Qualyclor solution | 10% |
|  | Silva [3] | Immersion in 25ppm Hidrosteril solution | 0% |
|  | Duhain et al. [1] | Immersion in 100ppm chlorine solution | 5% |
|  | Jesus et al. [2] | Immersion in commercial sanitizer solution (10ppm) | 10,2% |
|  | Nascimento et al. [6] | Immersion in 100ppm chlorine solution | 100% |
|  | Soares et al. [7] | Immersion in 100ppm chlorine solution | 100% |
| Chlorine solutions uninformed concentrations | Fernandes et al. [8] | Immersion in sodium hypochlorite (NI) solution | 71% |
|  | Fernandes et al. [8] | Immersion in chlorine solution (NI) | 8.7% |
| Detergents | Kadono et al. [9] | Solution with detergent | 87.5% |
|  | Silva [3] | Solution with neutral detergent | 10% |
|  | Soares et al. [10] | Sodium Lauryl Ether Sulphate + Ethoxylated Lauryl Alcohol | 94% |
|  | Sena et al. [11] | Cocostarchpropyl betaine 1:100 | 53% |
|  | Sena et al. [11] | Cocostarchpropyl betaine 1:1000 | 40% |
|  | Sena et al. [11] | Polyoxyethylene p-t-octylphenyl ether 1:100 | 37% |
|  | Sena et al. [11] | Polyoxyethylene p-t-octylphenyl ether 1:1000 | 26% |
|  | Sena et al. [11] | Sodium Lauryl Ether Sulfate 1:100 | 63% |
|  | Sena et al. [11] | Sodium Lauryl Ether Sulfate 1:1000 | 53% |
|  | Sena et al. [11] | Cetyl Trimethyl Ammonium Chloride 1:100 | 52% |
|  | Sena et al. [11] | Cetyl Trimethyl Ammonium Chloride 1:1000 | 37% |
|  | Sena et al. [11] | Ammonium Lauryl Ether Sulfate 1:100 | 77% |
|  | Sena et al. [11] | Ammonium Lauryl Ether Sulfate 1:1000 | 65% |
|  | Hajipour et al. [5] | 2 drops of detergent | 24.7% |
|  | Hafez et al. [12] | 15-25 drops of detergent | -5.9% |
|  | Higuti [13] | 3.6% sodium alkyl benzene sulfonate solution - 10 min | 66% |
|  | Higuti [13] | 3.6% sodium alkyl benzene sulfonate solution - 30 min | 47% |
| Saline solutions | Al Mozan et al. [14] | Immersion in 0.9% NaCl solution | 30% |
|  | Kudah et al. [15] | Immersion in 0.45% NaCl solution | -14% |
|  | Kudah et al. [15] | Immersion in 0.45% NaCl solution double wash | -7.5% |
|  | Kudah et al. [15] | Immersion in 0.9% NaCl solution | 2% |
|  | Kudah et al. [15] | Immersion in 0.9% NaCl solution double wash | 7.5% |
|  | Kudah et al. [15] | Immersion in 1.5% NaCl solution | 2% |
|  | Kudah et al. [15] | Immersion in 1.5% NaCl solution double wash | 20% |
|  | Amoah et al. [16] | Immersion in 7ppm NaCl solution | 89% |
|  | Amoah et al. [16] | Immersion in 23ppm NaCl solution | 67% |
|  | Amoah et al. [16] | Immersion in 35ppm NaCl solution | 60% |
|  | Woldetsadik et al. [17] | Immersion in 40 ppm NaCl solution | 72% |
| Acetic acid solution | El‐Trás et al. [18] | Immersion in 15,000ppm acetic acid solution/25°C | 60% |
|  | El‐Trás et al. [18] | Immersion in 15,000ppm acetic acid solution/45°C | 100% |
|  | Nascimento et al. [6] | Immersion in 4,400ppm acetic acid solution | 55% |
|  | Amoah et al. [16] | Immersion in 6,818-ppm acetic acid solution | 78% |
|  | Elahi et al. [19] | Immersion in 500ppm acetic acid solution | 85% |
|  | Silva [3] | Immersion in 500ppm acetic acid solution | 0% |
|  | Hajipour et al. [5] | Immersion in 500ppm acetic acid solution | 43% |
|  | Soares et al. [7] | Immersion in 400ppm acetic acid solution | 65% |
|  | Fernandes et al. [8] | Immersion in acetic acid solution (NI) | 100% |
|  | Jesus et al. [2] | Immersion in 800ppm acetic acid solution | 9% |
|  | Higuti [13] | Immersion in 15,000ppm acetic acid solution /10 min | 56% |
|  | Higuti [13] | Immersion in 15,000ppm acetic acid solution /30 min | 63% |
|  | Woldetsadik et al. [17] | Immersion in 15,000ppm acetic acid solution | 55% |
| Physical treatments | El‐Trás et al. [18] | Immersion in water at 25°C | 0% |
|  | El‐Trás et al. [18] | Immersion in water at 45°C | 40% |
|  | El‐Trás et al. [18] | Immersion in water at 65°C | 100% |
|  | Fallah et al. [20] | Water immersion | 96% |
|  | Mogharbel et al. [21] | Wash in water | 75% |
|  | Amoah et al. [16] | Wash in water | 89% |
|  | Elahi et al. [19] | Water immersion | 61% |
|  | Salavati et al. [4] | Water immersion | 23% |
|  | Woldetsadik et al. [17] | Wash in water | 64% |
|  | Fernandes et al. [8] | Water immersion | -5% |
|  | Craighead et al. [22] | Pulsed light on coriander | 100% |
|  | Craighead et al. [22] | Pulsed light on lettuce | 100% |
|  | Craighead et al. [22] | Pulsed light on spinach | 100% |
|  | Craighead et al. [22] | Pulsed light on tomatoes | 99% |
|  | Craighead et al. [23] | Cold plasma | 99% |
|  | Duhain et al. [1] | Freezing | 14% |
|  | Duhain et al. [1] | Microwave | 88% |
|  | Duhain et al. [1] | Bleaching | 88% |
|  | Kniel et al. [24] | High hydrostatic pressure (basil 10^4^ oocysts) | 100% |
|  | Kniel et al. [24] | High hydrostatic pressure (basil 10^6^ oocysts) | 100% |
|  | Kniel et al. [24] | UV light 254 on contamination 10^4^ | 100% |
|  | Kniel et al. [24] | UV light 254 on contamination 10^6^ | 37.5% |
| Others | Elahi et al. [19] | Uninformed germicide | 94% |
|  | Ortega et al. [25] | ClO_2_ in lettuce with *Crysptosporidium* spp | 100% |
|  | Ortega et al. [25] | ClO_2_ in lettuce with *Cyclospora* sp | 26.8% |
|  | Ortega et al. [25] | ClO_2_ in basil with *Crysptosporidium* spp | 100% |
|  | Ortega et al. [25] | ClO_2_ in basil with *Cyclospora* sp | -7% |
|  | Hajipour et al. [5] | Immersion in 1% lemon juice solution | 57% |
| Combined interventions | Immersion in 100ppm chlorine solution + brushing/rinsing | |  |
|  | Silva [3] | Immersion in Pury Vita solution (70 ppm) + washing + brushing | 0% |
|  | Silva [3] | Immersion in Useclor solution (80 ppm) + washing + brushing | 4% |
|  | Silva [3] | Immersion in Qualyclor solution (25 ppm) + washing + brushing | 98% |
|  | Silva [3] | Immersion in Hidrosteril solution (25ppm) + washing + brushing | 0% |
|  | Immersion in 200ppm chlorine solution + brushing/rinsing | |  |
|  | Costa [26]  Organic | Prewash + chlorine solution (200ppm) + rinse. | 91% |
|  | Costa [26]  Hydroponic | Prewash + chlorine solution (200ppm) + rinse. | 67% |
|  | Costa [26]  Conventional | Prewash + chlorine solution (200ppm) + rinse. | 88% |
|  | Fallah et al. [20] | Rinse under running water, soak in 200ppm calcium hypochlorite solution and rinse in automated equipment | 100% |
|  | Avcioglu et al. [27] | Rinse under running water, soak in 200ppm calcium hypochlorite solution (30m) and rinse in automated equipment | 100% |
|  | Kozan et al. [28] | Washing + immersion in 200ppm calcium hypochlorite solution + automated rinsing | 100% |
|  | Silva [3] | Immersion in bleach solution (250ppm) + washing + brushing | 96% |
|  | Soaking in detergent + chlorination + brushing/rinsing | |  |
|  | Beletini et al. [29] | Immersion in detergent (EXTRAN) solution + brushing + bleach (2.0 to 2.5%HclO) (200pppm) | 89% |
|  | Beletini et al. [29] | Immersion in detergent (EXTRAN) solution + brushing + HclO 1% | 89% |
|  | Rostami et al. [30] | Immersion in water + Immersion in water with detergent + Immersion in200ppm calcium hypochlorite solution + washing in running water | 100% |
|  | Costa [26]  Organic | Pre-wash+ Soaking in detergent solution (100ppm) + rinse + chlorine solution (200ppm) + rinse. | 96% |
|  | Costa [26]  Hydroponic | Pre-wash+ Soaking in detergent solution (100ppm) + rinse + chlorine solution (200ppm) + rinse. | 93% |
|  | Costa [26]  Conventional | Pre-wash+ Soaking in detergent solution (100ppm) + rinse + chlorine solution (200ppm) + rinse. | 94% |
|  | Yarahmadi et al. [31] | Rinse + immersion in water and 3-5 drops of detergent + immersion in 200ppm calcium hypochlorite solution + washed in running water | N/A |
|  | Immersion in Vinegar + brushing/rinsing | |  |
|  | Beletini et al. [29] | Immersion in detergent (EXTRAN) solution + brushing + 40% acetic acid | 68% |
|  | Soares et al. [10] | Immersion in 5,400ppm acetic acid solution + washing in running water and immersion in distilled water | 99% |
|  | Silva [3] | Immersion in 500ppm Vinegar solution + washing + brushing | 0% |
|  | Immersion in Vinegar + saline + brushing/rinsing | |  |
|  | Al Mozan et al. [14] | Wash with saline solution + brushing + vinegar (5% Acetic Acid) + immersion in water | 70% |
|  | Amoah et al. [16] | Washing with saline solution (7 ppm)/vinegar (6,818 ppm) | 67% |
|  | Woldetsadik et al. [17] | Washing with water + immersion in vinegar (15000ppm acetic acid) and 40ppm saline | 72% |
|  | Others | |  |
|  | Duhain et al. [1] | 200ppm Chlorine + Blast freezing | 0% |
|  | Duhain et al. [1] | 200ppm Chlorine +Microwave | 92% |
|  | Soares et al. [10] | Immersion in sodium hypochlorite solution + permanganate + washing in running water and immersion in distilled water | 100% |
|  | Silva [3] | Soaking in detergent solution + washing + brushing | 100% |

**References**

1. Duhain GL, Minnaar A, Buys EM. Effect of chlorine, blanching, freezing, and microwave heating on Cryptosporidium parvum viability inoculated on green peppers. J Food Prot. 2012;75 (5):936-41. doi:10.4315/0362-028X.JFP-11-367
2. Jesus NAC, Macedo ME. Avaliação dos sanitizantes para eliminação dos ovos de Toxocara canis em alface (Lactuca sativa L.). Centro Universitário Metodista Izabela Hendrix.2014: Acervo Iniciação Científica.1
3. Silva APDR. Avaliação da eficácia dos desinfetantes para controle de larvas de nematoda em hortaliças. [B.Sc thesis]. Brasília: Universidade de Brasília, 2017. Available from: https://bdm.unb.br/bitstream/10483/18441/1/2017_AnaPereiradaRochaSilva_tcc.pdf
4. Salavati Z, Chalehchaleh AA, Rezaei F. Parasitic Infections in Raw Vegetables of Kermanshah, Western Iran and Their Relation with Season and Washing Procedures. J Food Qual Hazards Control.2017; 4 (2):37–41.
5. Hajipour N, Soltani M, Ketzis J, Hassanzadeh P. Zoonotic parasitic organisms on vegetables: Impact of production system characteristics on presence, prevalence on vegetables in northwestern Iran and washing methods for removal. Food Microbiology.2021;95 (103704).doi:10.1016/j.fm.2020.103704
6. Nascimento ED do, Alencar FLS. Eficiência antimicrobiana e antiparasitária de desinfetantes na higienização de hortaliças na cidade de Natal - RN. Ciência e Nat. 2014;36(2):92–106. doi:10.5902/2179460X12755
7. Soares EF. Eficácia microbiana e parasitária de sanitizantes à base de cloro e ácido acético em alface (Lactuca sativa L.). [B.Sc thesis]. Cruz Alta: Universidade Estadual do Rio Grande do Sul, 2019,16f. Available from: https://repositorio.uergs.edu.br/xmlui/bitstream/handle/ 123456789/1019/2_artigo_eveline.pdf?sequence=-1&isAllowed=y
8. Fernandes NS, Guimarães HR, Amorim ACS, Reis MB. Avaliação parasitológica de hortaliças: da horta ao consumidor final. Saúde e Pesquisa. 2015;8(2):255-265.doi:10.17765/1983-1870.2015v8n2p255-265
9. Kadono Y, Kashiwagi Y, Shibata M, Tokue S, Ohkubo N. Effect of synthetic detergents on the elimination of bacteria and Ascaris eggs from vegetables. Annual Report of Tokyo Metropolitan Research Laboratory of Public Health.1973; 24, 47-52
10. Soares B, Cantos GA. Detecção de estruturas parasitárias em hortaliças comercializadas na cidade de Florianópolis, SC, Brasil. Rev Bras Ciencias Farm J Pharm Sci. 2006;42(3):455–60. doi:10.1590/S1516-93322006000300015
11. Sena A. Avaliação de agentes tensoativos na descontaminação de ovos de Ascaris sp. em amostras da hortaliça Lactuca sativa. [Dissertation]. Rio Grande: Universidade Federal do Rio Grande, 2007. Available from: https://www2.ufpel.edu.br/cic/2006/arquivos/CS_00447.rtf
12. Hafez AA, Asadolahi E, Havasian M, Anahi J, Davoudian A, Lotfikir M, Khosravi A. Study on the parasitic and microbial contamination of vegetables, and the effect of washing procedures on their elimination in Ilam city. J Paramed Sci.2013; 4(4):37–41
13. Higuti STM. Efeito do vinagre e detergente doméstico na remoção de cistos de Giardia duodenalis em folhas de alface crespa (Lactuca sativa). [Dissertation]. Curitiba: Universidade Federal do Paraná; 2009. 39f. Available from: https://acervodigital.ufpr.br/bitstream/handle/ 1884/30363/Monografia%20Silvia%20Tieme%20Makita%20Higuti.pdf?sequence=1&isAllowed=y
14. Al-Mozan HD, Dakhil KM. Prevalence of Parasites in Fresh Vegetables from Two Regions of Thi-Qar Province, Iraq. Journal of Pure and Applied Microbiology. 2019;13(2),1103-1110. doi:10.22207/JPAM.13.2.49
15. Kudah C, Sovoe S, Baiden F. Parasitic contamination of commonly consumed vegetables in two markets in Ghana. Ghana Med J. 2018;52(2):88–93. doi:10.4314/gmj.v52i2.5
16. Amoah P, Drechsel P, Abaidoo RC, Klutse A. Effectiveness of common and improved sanitary washing methods in selected cities of West Africa for the reduction of coliform bacteria and helminth eggs on vegetables. Trop Med Int Health. 2007;12 Suppl 2:40-50. doi:10.1111/j.1365-3156.2007.01940.x
17. Woldetsadik D, Drechsel P, Keraita B, Itanna F, Erko B, Gebrekidan H. Microbiological quality of lettuce (Lactuca sativa) irrigated with wastewater in Addis Ababa, Ethiopia and effect of green salads washing methods. Food Contamination.2017;4(3). doi:10.1186/s40550-017-0048-8
18. El-Trás WF, Tayel AA, El-Kady NN. Source Diversity Of Toxoplasma Gondii Infection During Meal Preparation. Journal of Food Safety. 2011;32:1-5. doi:10.1111/j.1745-4565.2011.00336.x
19. Elahi R, Kheirabadi YP, Ahmadi N, Gholamalizade M, Dehkodi HA. The Effect of Washing Procedures on Contamination of Raw Vegetables with Nematodes Larvae. Asian Journal of Pharmaceutics. 2018;12(02). doi:10.22377/ajp.v12i02.2381.
20. Fallah AA, Pirali-Kheirabadi K, Shirvani F, Saei-Dehkordi SS. Prevalence of parasitic contamination in vegetables used for raw consumption in Shahrekord, Iran: Influence of season and washing procedure. Food Control. 2012;25(2):617-620. doi:10.1016/j.foodcont.2011.12.004
21. Mogharbel AD, Reis F, Masson ML. Survey of biological hazards in the lettuce used in commercial snacks (sandwiches) from Curitiba, PR, Brazil. Alimentos e Nutrição. 2008;19(3): 235-241
22. Craighead S, Huang R, Chen H, Kniel KE. The use of pulsed light to inactivate Cryptosporidium parvum oocysts on high-risk commodities (Cilantro, mesclun lettuce, spinach, and tomatoes). Food Control.2021;126.doi:10.1016/j.foodcont.2021.107965.
23. Craighead S, Hertrich S, Boyd G, Sites J, Niemira BA, Kniel KE. Cold Atmospheric Plasma Jet Inactivates Cryptosporidium parvum Oocysts on Cilantro. J Food Prot. 2020; 83(5):794-800. doi:10.4315/0362-028X.JFP-19-442
24. Kniel KE, Shearer AE, Cascarino JL, Wilkins GC, Jenkins MC. High hydrostatic pressure and UV light treatment of produce contaminated with Eimeria acervulina as a Cyclospora cayetanensis surrogate. J Food Prot. 2007;70(12):2837-42. doi:10.4315/0362-028x-70.12.2837
25. Ortega YR, Mann A, Torres MP, Cama V. Efficacy of gaseous chlorine dioxide as a sanitizer against Cryptosporidium parvum, Cyclospora cayetanensis, and Encephalitozoon intestinalis on produce. J Food Prot. 2008;71(12):2410-4. doi:10.4315/0362-028x-71.12.2410
26. Costa EA. Avaliação microbiológica e parasitológica nos processos de higienização de alfaces (Lactuca sativa L.) de diferentes cultivos. [Dissertation]. Fortaleza: Universidade do Ceará; 2011. Available from: https://repositorio.ufc.br/bitstream/riufc/17438/1/20011_dis_eacosta.pdf
27. Avcioglu H, Soykan E, Tarakci U. Control of Helminth Contamination of Raw Vegetables by Washing. Vector-borne and Zoonotic Diseases. 2011;11(2):189-191. doi:10.1089=vbz.2009.0243
28. Kozan E, Gonenc B, Sarimehmetoglu O, Aycicek H. Prevalence of helminth eggs on raw vegetables used for salads. Food Control.2005;16:239–242. doi: 10.1016/j.foodcont.2004.02.005.
29. Beletini LF, Takizawa LHH, Takizawa MG. Enteroparasitas em alfaces (Lactuca sativa) variedade crespa previamente tratadas com desinfetantes. Revista Thêma et Scientia.2014; 4(1): 150-157
30. Rostami A, Ebrahimi M, Mehravar S, Fallah Omrani V, Fallahi S, Behniafar H. (2016). Contamination of commonly consumed raw vegetables with soil transmitted helminth eggs in Mazandaran province, northern Iran. Int J Food Microbiol.2016;225:54–8. doi:10.1016/j.ijfoodmicro.2016.03.013.
31. Yarahmadi M, Yunesian M, Pourmand M, Shahsavani A, Mubedi I, Nomanpou B, Naddafi K. Evaluating the efficiency of lettuce disinfection according to the official protocol in Iran. Iran J Public Health. 2012;41(3):95-103.
