# Supplementary figures and images for: Effectiveness of sanitization protocols in removing or reducing parasites from vegetables: A systematic review with meta-analysis
Source: PLoS One. 2023 Sep 1;18(9):e0290447. doi: 10.1371/journal.pone.0290447 (PMC10473522; doi:10.1371/journal.pone.0290447)

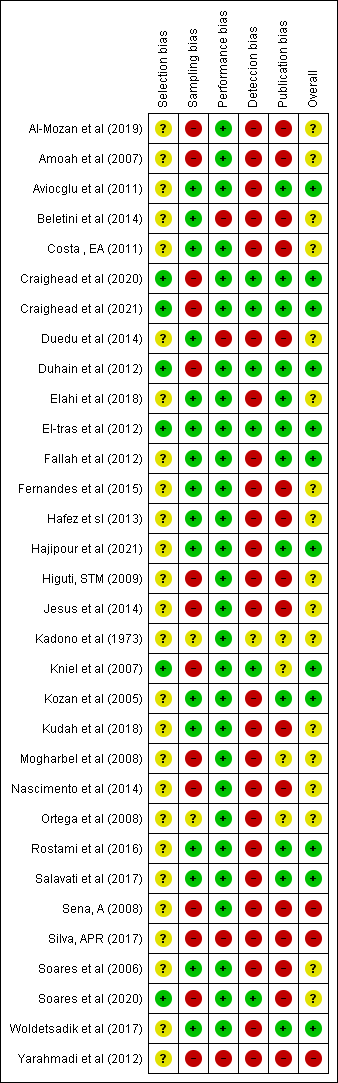

Supplement: S5 File — (PNG) [file pone.0290447.s005.png]
