# Supplementary material for: Effectiveness of sanitization protocols in removing or reducing parasites from vegetables: A systematic review with meta-analysis
Source: PLoS One. 2023 Sep 1;18(9):e0290447. doi: 10.1371/journal.pone.0290447 (PMC10473522; doi:10.1371/journal.pone.0290447)

**Supplementary Material 6 - Analysis of subgroups and sensitivity**

1. **Meta-analyses of chlorine solutions**

Figure 1. Forest plot of interventions with chlorine solutions


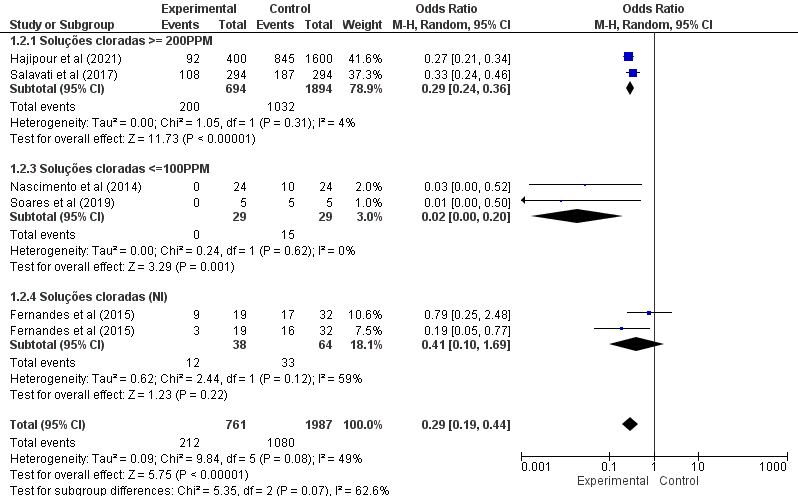


It can be seen in **Figure 1** that there was moderate heterogeneity (I^2^=49%) between studies and the final sanitizing effect was 0.29 OR [95%CI 0.19; 0.44], meaning that vegetables after sanitization with chlorinated solutions have a 71% lower chance of not finding parasites compared to unsanitized vegetables.

Figure 2. Forest plot of interventions with chlorinated solutions with artificial contamination.


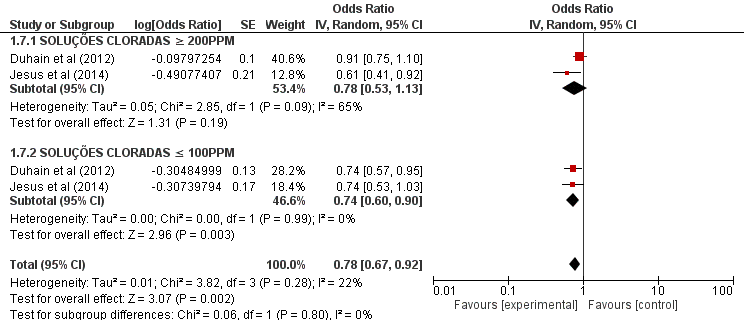


The heterogeneity was lower (I^2^=22%)in **Figure 2**, but with a weaker sanitizing effect 0.78 OR [95%CI 0.67; 0.92], which means a potential for sanitizing vegetables of only 22% when compared to control samples.

For the sensitivity analysis, studies with more than 02 high-risk points for the bias assessment were removed from the meta-analysis. **Figure 3** shows the sensitivity analysis excluding the study by Nascimento et al., and the result presented in the sensitivity analysis demonstrates that the meta-analysis was not affected by the modification of studies, attributing a greater degree of certainty regarding the outcome found.

Figure 3. Sensitivity analysis of interventions with chlorine


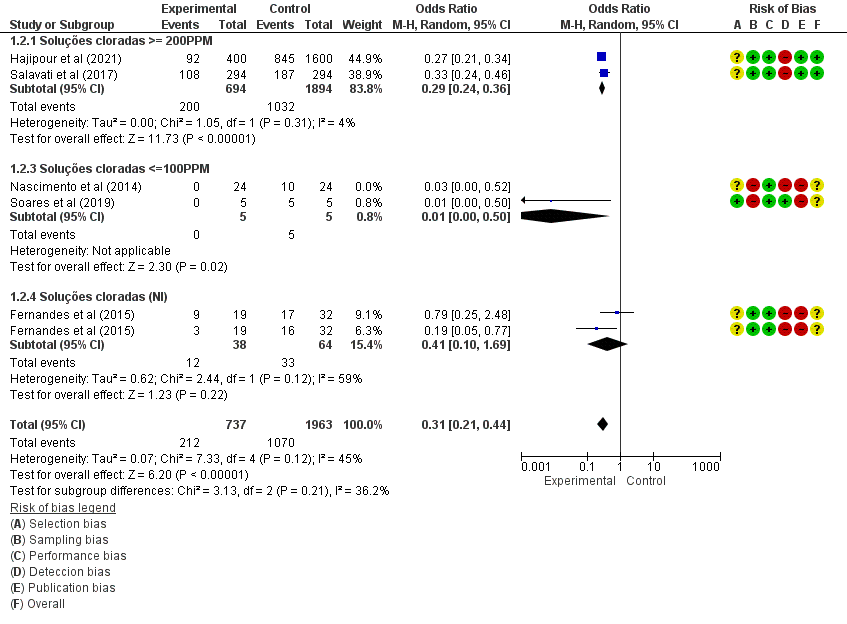


1. **Meta-analyses of detergent solutions**

Figure 4. Graph of interventions with detergents.


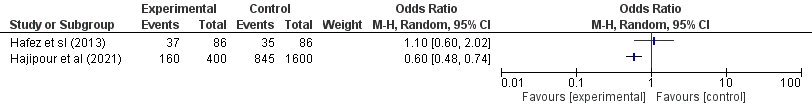


Figure 5 Graph of interventions with detergents in artificial contamination.


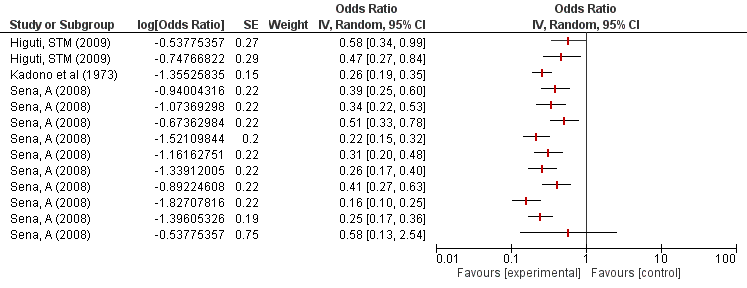


Meta-analyses of studies with detergent interventions showed substantial heterogeneity above 50%, and the OR value was not considered valid, as the greater the heterogeneity, the greater the questioning about the validity of combining these results. Therefore, a sensitivity analysis was not performed.

1. **Meta-analyses of saline solutions**

Figure 6. Forest plot of interventions with saline solutions.


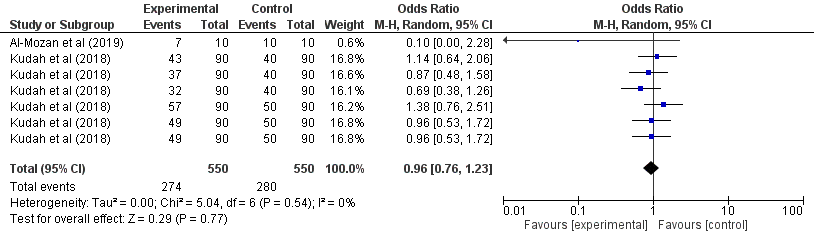


The heterogeneity of the outcomes included in the graph in **Figure 6** was low, with values of p>0.10 and I^2^=0 (Heterogeneity: Chi² = 5.04, df = 6 (P = 0.54); I² = 0%), it is possible to note that the lower diamond that represents the synthesis of these studies exceeded the central trend line, and therefore there was no evidence that this type of treatment is sufficient to remove parasites in the foods tested. There were no studies with artificial contamination in this type of intervention.

For the sensitivity analysis, shown in **Figure 18**, the study by Al-Mozan et al. which had three high values for the assessment of bias, and the odds ratio value maintained the result found in the meta-analysis, however, as these are data from a single study, a high level of reliability cannot be attributed to this evidence.

Figure 7. Sensitivity analysis of interventions with saline solutions.


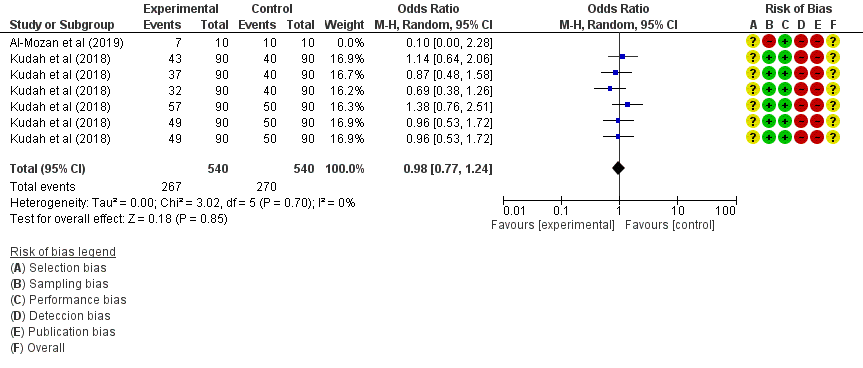


1. **Meta-analyses of acetic acid solutions**

Figure 8. Graph of interventions with acetic acid solutions.


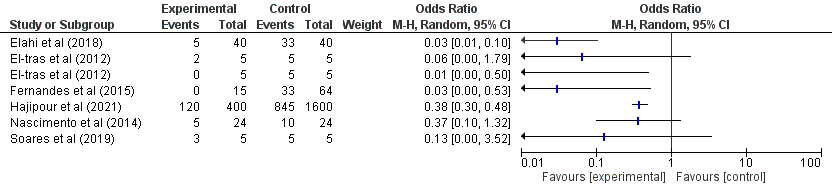


Figure 9. Graph of interventions with acetic acid solutions with artificial contamination


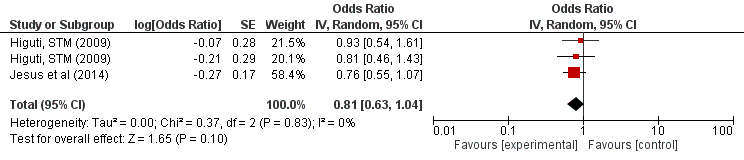


The meta-analyses of studies with interventions using acetic acid solutions showed a high heterogeneity, above 50%, and the OR value was not considered valid, nor was a sensitivity analysis performed.

1. **Meta-analyses of the physical treatments**

Figure 10. Graph of the interventions with rinses or immersion in water.


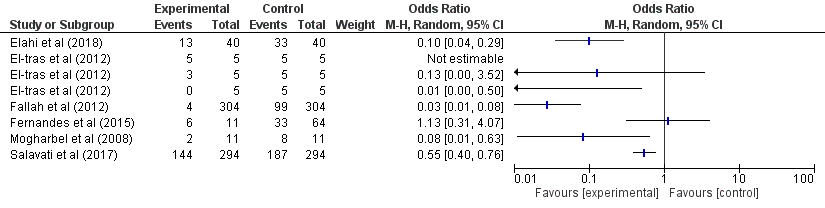


Figure 11. Graph of the other physical interventions.


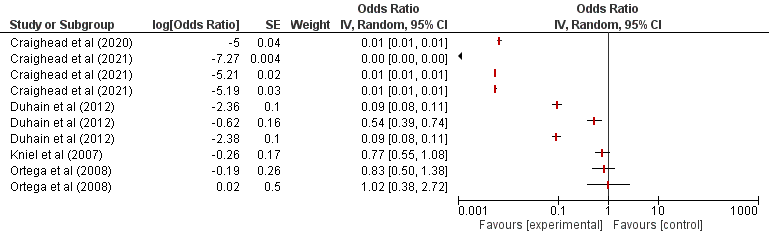


The meta-analyses of studies with interventions through physical treatments also showed a high heterogeneity, above 50%, and the OR value was not considered valid, and therefore the sensitivity analysis was not performed.

**6. Meta-analyses of the combined interventions**

Figure 12. Forest plot of the combined interventions.


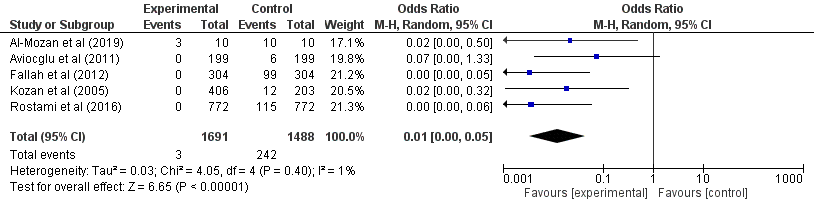


Figure 13. Forest plot of interventions combined by inverse of variance.


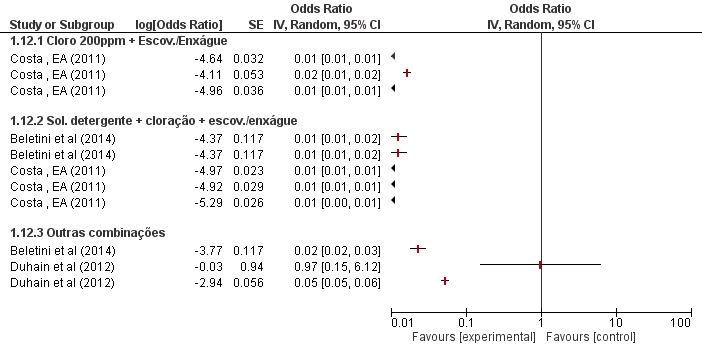


**Figure 12** represents the forest plot of the combined interventions and shows a very low heterogeneity with I^2^ of 1%, but with Chi^2^ just above 0.10 (Heterogeneity: Chi² = 4.05, df = 4 (P = 0.40); I² = 1%). The final odds ratio value shows a very low result of 0.01OR [95%CI: 0.00 – 0.05], meaning that there is an estimated 99.99% lower chance of finding parasites among the selected combined interventions than in relation to unsanitized vegetables, conferring the greatest protective effect among the combinations of treatments.

**Figure 13** shows the other outcomes that could not be calculated by the first graph, in which it is possible to observe that most studies have very favorable results, with odds ratio close to the values found in **Figure 12**; however, the heterogeneity between studies was too high (Heterogeneity: Tau² = 0.41; Chi² = 51.54, df = 2 (P < 0.00001); I² = 96%), and therefore we do not calculate a valid OR result.

A sensitivity analysis was performed to assess the robustness of the first result, taking the study by Al-Mozan et al. which had three results at high risk of bias. **Figure 14** shows the graph of the sensitivity analysis demonstrating that there was no change in the outcome, meaning that the result was not affected by the presence of studies with greater variability, and this result is considered with a greater degree of certainty.

Figure 14. Sensitivity analysis of combined interventions


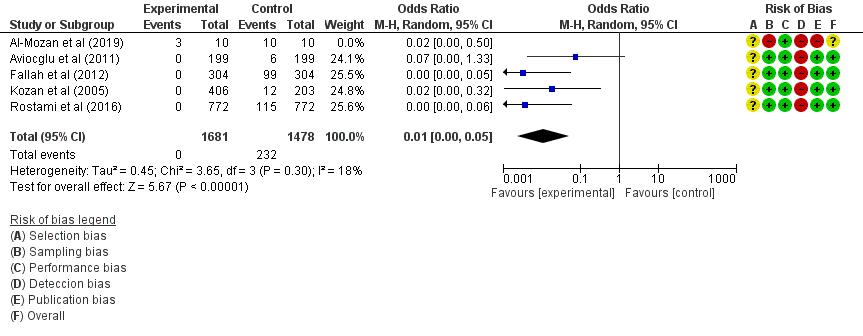

Supplement: S6 File — (DOCX) [file pone.0290447.s006.docx]
