# Supplementary material for: Effectiveness of sanitization protocols in removing or reducing parasites from vegetables: A systematic review with meta-analysis
Source: PLoS One. 2023 Sep 1;18(9):e0290447. doi: 10.1371/journal.pone.0290447 (PMC10473522; doi:10.1371/journal.pone.0290447)
Supplement: S7 File — (DOCX) [file pone.0290447.s007.docx]

# Supplementary Material 7 – GRADE evaluation

| **Efficacy of parasitic decontamination compared to non-intervention for removing parasites in vegetables: a systematic review with meta-analysis** | | | | | | |
| --- | --- | --- | --- | --- | --- | --- |
| **Patient or population:** Vegetables  **Intervention:** Efficacy of parasitic decontamination  **Comparison:** Non-intervention | | | | | | |
| Outcomes | **Anticipated absolute effects^*^**(95% CI) | | Relative effect (95% CI) | № of participants (studies) | Certainty of the evidence (GRADE) | Comments |
|  | **Risk with non-intervention** | **Risk with Efficacy of parasitic decontamination** |  |  |  |  |
| Chlorine solutions≥ 200ppm | 545 per 1,000 | **258 per 1,000** (223 to 301) | **OR 0.29** (0.24 to 0.36) | 2588 | ⨁⨁⨁◯ Moderate^a^ |  |
| Chlorine solutions≤ 100ppm | 516 per 1,000 | **138 per 1,000** (31 to 470) | **OR 0.15** (0.03 to 0.83) | 160 | ⨁◯◯◯ Very low ^b,c,d^ |  |
| Detergents | 522 per 1,000 | **453 per 1,000** (314 to 598) | **OR 0.76** (0.42 to 1.36) | 2172 | ⨁◯◯◯ Verylow^b,c^ |  |
| Saline solutions | 509 per 1,000 | **499 per 1,000** (441 to 561) | **OR 0.96** (0.76 to 1.23) | 1100 | ⨁⨁◯◯ Low^b^ |  |
| Acetic acid solutions | 533 per 1,000 | **138 per 1,000** (33 to 386) | **OR 0.14** (0.03 to 0.55) | 2207 | ⨁◯◯◯ Verylow^b,c^ |  |
| Immersion in water | 505 per 1,000 | **155 per 1,000** (39 to 433) | **OR 0.18** (0.04 to 0.75) | 1373 | ⨁◯◯◯ Verylow^a,e^ |  |
| Combined interventions | 163 per 1,000 | **2 per 1,000** (0 to 10) | **OR 0.01** (0.00 to 0.05) | 3179 | ⨁⨁◯◯ Low^a,c^ |  |
| ***The risk in the intervention group** (and its 95% confidence interval) is based on the assumed risk in the comparison group and the **relative effect** of the intervention (and its 95% CI).  **CI:** confidence interval; **OR:** odds ratio | | | | | | |
| **GRADE Working Group grades of evidence**  **High certainty:** we are very confident that the true effect lies close to that of the estimate of the effect.  **Moderate certainty:** we are moderately confident in the effect estimate: the true effect is likely to be close to the estimate of the effect, but there is a possibility that it is substantially different.  **Low certainty:** our confidence in the effect estimate is limited: the true effect may be substantially different from the estimate of the effect. **Very low certainty:** we have very little confidence in the effect estimate: the true effect is likely to be substantially different from the estimate of effect. | | | | | | |

Explanations

a. Downgraded in one level due to high risk detection bias in the included studies.

b. Downgraded in two levels due to high risk detection and publication bias in the included studies.

c. Downgraded in one level due to inconsistency.

d. Downgraded in one level due to imprecision (small sample size and wide CI)

e. Downgraded in two levels due to inconsistency.
